# Reconstructing human history from ancient genomes: an interview with Nobel laureate Svante Pääbo

**DOI:** 10.1093/nsr/nwae120

**Published:** 2024-03-27

**Authors:** Weijie Zhao

**Affiliations:** NSR Based, Beijing

## Abstract

*Professor Svante Pääbo, Director of the Max Planck Institute for Evolutionary Anthropology, won the Nobel Prize in Physiology or Medicine in 2022 for his discoveries in ancient hominine genomes and human evolution. His pioneering work in sequencing and interpreting the paleo genomes of Neanderthals and Denisovans, as well as their relationship with the modern human genome, was groundbreaking in terms of our understanding of human origins. Nowadays, we can even use commercial kits to easily detect the proportion of Neanderthal genes in our own genomes*.

*Recently, *NSR* conducted an interview with Professor Pääbo to learn about his interesting work chasing the ancient genomes and reconstructing human evolutionary and migration history from the DNA evidence, as well as his perspective on paleo genome studies and his advice to young researchers: follow your interests and be ready to try some crazy things*.

## Teaser

NSR's interview with 2022 Nobel laureate Svante Pääbo to learn about his interesting works in ancient hominine genomes, his perspectives on paleo genome studies and his advice to young researchers.

***NSR:*** Your study of ancient DNA started with mummies. Are you still interested in these kinds of dried samples?

***Pääbo:*** These days, we are focused on DNA from extinct forms of humans such as Denisovans and Neanderthals and early modern humans, mostly working with bones but also with sediments from archaeological sites. Other researchers are working on mummified remains from Egypt and elsewhere. It turns out that it is often very difficult to retrieve DNA from such remains, and the very first DNA sequences I retrieved back in the early 1980s were surely contaminations from present-day people.

***NSR:*** Is it easier to retrieve DNA from bones/sediments than from mummies?

***Pääbo:*** In fact, our understanding of what factors determine the preservation of DNA over long periods is very limited. Acid conditions are bad, adsorption to minerals may help, but other factors that we are not aware of clearly play important roles.

## RECONSTRUCT THE HISTORY OF HUMAN EVOLUTION

***NSR:*** Your group made many discoveries related to the paleo genomes of Neanderthals and Denisovans. Currently, how much do we know about their genomes? What lies in the future?

***Pääbo:*** We have a few high-quality genomes of Neanderthals and still only one genome of a Denisovan. These genomes are of high quality in the sense that, for all parts of the genome to which we can match the short DNA fragments from ancient bones, we have seen every position on average 30 times or more. This means that we can determine, for example, what variants an individual inherited from just one parent. We have a much less clear picture of sequences that occur many times in the genome, as we cannot match the short fragments to them. For example, we can only have a statistical picture of how many copies of a certain repetitive sequence are present.

**Figure 1:**
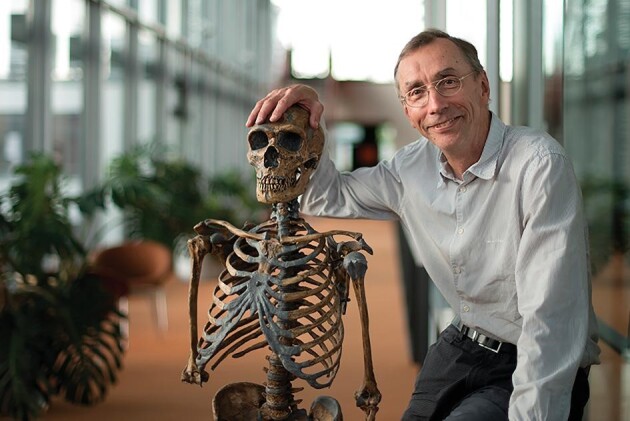
Svante Pääbo with a replicate Neandertal skeleton. (Credit: Karsten Möbius)

We also have a larger number of Neanderthal genomes of lower quality. This has allowed us to reconstruct some main aspects of their history. In the future, we will clearly retrieve many more genomes and learn more about their history and hopefully also about their biology. For example, it would be fascinating to learn more about how they may have differed from present-day people in terms of their physiology.

***NSR:*** Please briefly introduce the most recent model of human evolution and migration.

***Pääbo:*** Modern humans evolved in Africa and spread from there to the rest of the world. In that process they mixed with other earlier forms of humans that existed in Eurasia, particularly Neanderthals in western Eurasia and Denisovans in eastern Eurasia. As a result of that, one or two percent of the genomes of people whose roots are outside of Africa come from Neanderthals, and in Asia, people in addition carry genetic contributions from Denisovans. This general model is in my view accepted by most researchers today.

**Figure 2:**
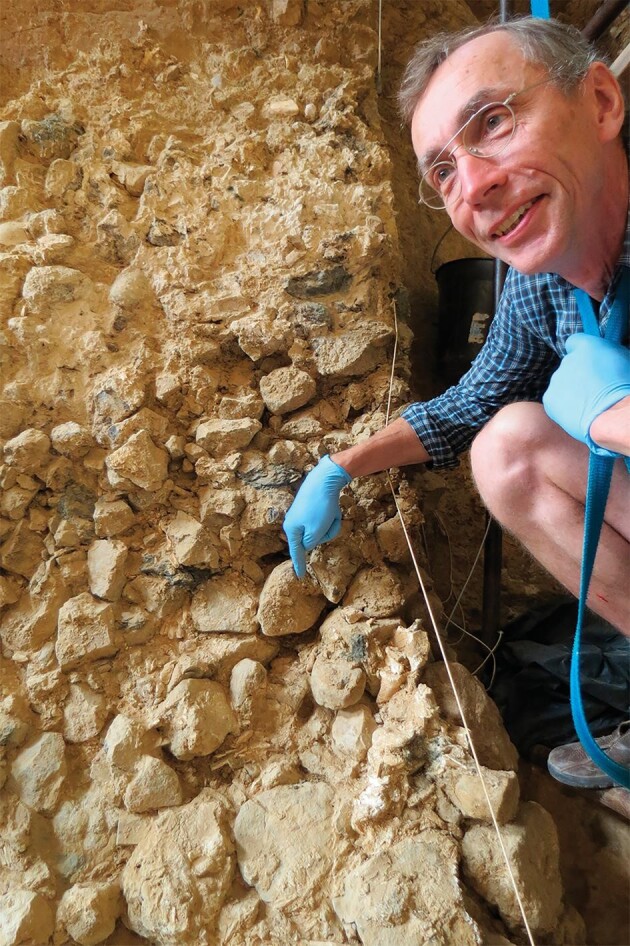
Svante Pääbo showing the location of a sediment sample collected at the site of Caune de l'Arago, France, from a layer dated to 450 000 years ago. *(Courtesy of Christian Perrenoud.)*

***NSR:*** What's your view on the debate between the Out-of-Africa model and the Multiregional Evolution model?

***Pääbo:*** The genomes of these extinct human groups have shown that a strict Out-of-Africa model for modern humans is not correct in that there have been contributions from earlier forms of humans to people who live today. However, a strict Multiregional model is also not correct, in that the vast majority of the genetic variations in present-day people come out of Africa quite recently in evolutionary terms.

***NSR:*** Why did modern humans ‘win’ and why did Neanderthals, Denisovans and other ancient human populations become extinct? What have we learnt from the genomes?

***Pääbo:*** That is one of the big questions to which we do not have a definitive answer. One interesting observation is that quite a lot of modern humans lived before 40 000 years ago. That is a time when Neanderthals were around. They have turned out to have Neanderthal relatives a few generations back in their family trees. This makes us think that one reason why Neanderthals disappeared may simply have been that modern humans were more numerous so that when they mixed with the less numerous Neanderthals, the latter ‘disappeared’. Clearly the answer will be more complex, but that may well be one major reason for why modern humans replaced the other groups. The outstanding question is then why modern humans were more numerous.

… modern humans were more numerous [so] when they mixed with the less numerous Neanderthals, the latter ‘disappeared’.

—Svante Pääbo

## PALEO GENOMICS STUDY

***NSR:*** What is the most difficult part of paleo genome sequencing? Is it the control of contamination? Has this problem been broadly solved yet?

***Pääbo:*** One major problem is indeed contamination. To a large extent, we know how to deal with it today, by laboratory precautions, by examining chemical modifications that accumulate over time in the DNA, and by other approaches. Another difficult area is how to best extract and manipulate the often very tiny amounts of DNA that survive in ancient specimens. In this area, there is still room for improvement.

**Figure 3:**
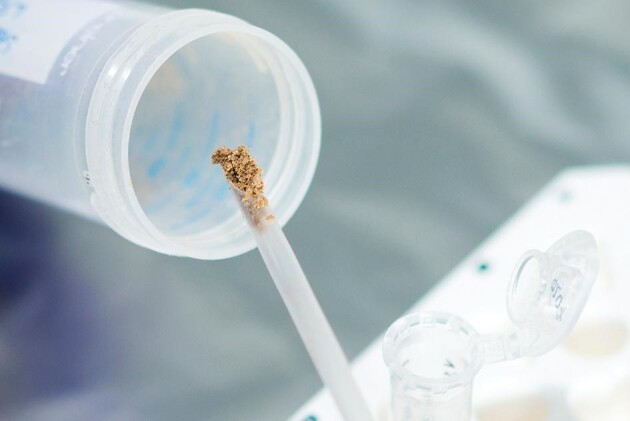
Researcher Viviane Slon preparing a sediment sample for DNA extraction. *(Courtesy of Sylvio Tüpke.)*

***NSR:*** What may be the next breakthrough in the field?

***Pääbo:*** We have many fossils of Neanderthals and they are restricted to western Asia and Europe. In contrast, we have hardly any Denisovan fossils, so we do not know much about where they lived or what they looked like. But they are likely to have been widespread in Asia, as they have contributed to the

ancestors of all people who live in Asia today. A likely place where Denisovan fossils may be found is China. Perhaps there are even fossils today in the museums in China that are actually Denisovans.

***NSR:*** What about studies of the genomes of non-human ancient animals? Are there any interesting recent discoveries?

***Pääbo:*** There are very many interesting studies by many groups, for example of mammoths and horses in the permafrost that go very far back in time, and of the history of brown bears and polar bears. Together with studies of plant and animal DNA in archaeological sediments, such studies can throw light on how environments and ecosystems changed over time.

Ancient DNA from domesticated animals and plants is also of great interest and tell us how past human populations domesticated different animal species and how these species then spread among human populations.

***NSR:*** Paleontologists are often good storytellers, but sometimes, different stories can be told based on the same evidence. What's your comment about this issue?

***Pääbo:*** I would only say that what we have always tried to do is to be very clear about what the data at hand actually show, and what aspects are interpretation or even speculation. But of course, we should not forget that our understanding of the past will never be complete and that future discoveries may overturn our current understanding.

## OTHER QUESTIONS

***NSR:*** What changes has the Nobel Prize brought to your life and research?

***Pääbo:*** A major change is the huge number of invitations and proposals for activities I get almost every day. It makes me feel bad to turn down so many generous and interesting invitations. Apart from that, I do my best to continue my life and research as it was before this happened.

***NSR:*** What are your current research interests? Are there any interesting discoveries that can be shared?

***Pääbo:*** A dream would be to understand some aspects of how modern humans differ from Neanderthals and Denisovans in a functional sense. We are working on understanding, using mouse and cell systems, the biological impact of some of the genetic changes that occurred in the ancestors of modern humans, and which are shared by many or all people today.

***NSR:*** It seems that you are very good at conducting research even when conditions are not what others would consider sufficient. One example is that you performed your mummy study in your PhD lab without telling your supervisor. Is this ability one of the keys to your success?

***Pääbo:*** I do not know. One good idea may be to be ready to try some crazy things. Many of those attempts will fail but sometimes you are lucky and you find something you can continue to work on. Of course, this has to be balanced with other projects, which are more likely to yield results.

***NSR:*** Please give some advice to young researchers.

***Pääbo:*** This is a hard question for me, which I get often these days. One piece of advice would be to follow your interests. If you are interested in what you do, you at least have a good time when you work on it, and you are generally good at things you like to do.

***NSR:*** Do you have collaborations with Chinese scientists?

***Pääbo:*** We have a long-standing collaboration with the Institute of Vertebrate Paleontology and Paleoanthropology (IVPP) of the Chinese Academy of Sciences that started thanks to a Chinese student, Qiaomei Fu (付巧妹), who did a PhD with us. She was a very talented student and is now a very talented researcher. We helped her and colleagues at the IVPP to start an ancient DNA lab and did some great projects with them.

## Notes

Weijie Zhao is a science and news editor for NSR based in Beijing.

